# PREDICTORS OF INTENT TO USE DIGITAL MEMORY BOOK AMONG DEMENTIA CAREGIVERS

**DOI:** 10.1093/geroni/igae098.4346

**Published:** 2024-12-31

**Authors:** Yura Lee, Chorong Oh, Min Sook Park, Ji Yae Bong

**Affiliations:** University of Wisconsin-Milwaukee, Milwaukee, Wisconsin, United States; Ohio University, Athens, Ohio, United States; Florida State University, Florida State University, Florida, United States; University of North Carolina at Charlotte, Charlotte, North Carolina, United States

## Abstract

A memory book is a popular reminiscence approach for people with dementia (PWD) and caregivers (CGs). Recently, it has evolved into a digital form by incorporating music, videos, or other multimedia, referred to as a digital memory book (DMB). Like conventional memory books, DMBs have been found to improve quality of life, cognition, communication, and mood for PWD and CGs, while also reducing CG burden. Despite these benefits, the usage of DMBs remains low, and there is limited research identifying factors influencing intent to use DMBs in this population. Considering that family CGs are most often the facilitators of DMBs for PWD, this study aims to examine the predictors of intent to use DMB among dementia CGs. Based on the Technology Acceptance Model, we collected data from 170 dementia CGs. Findings from linear regression analyses show that dementia type, primary caregiver status, age, race, education, caregiver health, and relationship to PWD were significantly related to intent to use DMBs. We also found a significant interaction effect between dementia type (Alzheimer’s disease [AD] vs. other dementias) and stage (mild vs. moderate to severe) (b=0.493, SE=0.217, p <.05). Specifically, differences in intent to use DMBs between mild and moderate to severe stages were significantly greater among CGs for AD compared to other dementias (ΔM= 0.45 vs. 0.05). This study contributes to identifying predictors of the intent to use DMB among dementia CGs. Considering these factors will be crucial in developing and implementing DMB programs to enhance their use, thereby benefiting this population.

